# Public health round-up

**DOI:** 10.2471/BLT.20.010420

**Published:** 2020-04-01

**Ebola outbreak winds down**

A WHO Ebola vaccination team working in the city of Butembo on 01 January 2019. The commitment and hard work of health professionals has been crucial in tackling the Ebola virus disease outbreak which was first declared in the Democratic Republic of the Congo in 2018 and now appears to be winding down.

**Figure:**
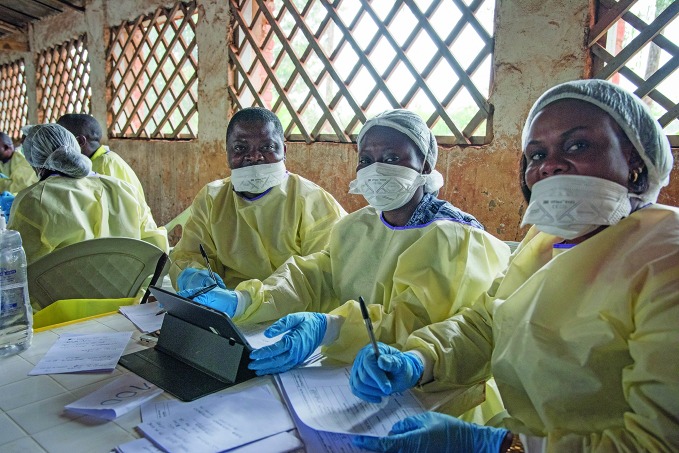
WHO/Lindsay Mackenzie

## WHO names COVID-19 a pandemic

World Health Organization (WHO) Director-General Tedros Adhanom Ghebreyesus declared the COVID-19 outbreak to be a pandemic. Speaking at a media briefing on 11 March, Dr Tedros said that while the term pandemic can cause unreasonable fear, or unjustified acceptance that the fight is over, the organization had decided to use it in response to the “alarming levels of spread and severity (of COVID-19), and by the alarming levels of inaction.”

Describing the COVID-19 outbreak as a pandemic does not change WHO’s assessment of the threat posed by the virus or WHO’s response activities. Nor does it change what countries should do. Dr Tedros concluded by underlining the fact that the pandemic can be controlled if countries implement strategies to detect, test, treat and isolate those people infected.

As of 13 March, 125 048 people were confirmed to be infected with COVID-19, 4613 of whom had died. Most cases were in China (80 981 confirmed) as were most deaths (3173). Outside of China, 44 067 cases had been confirmed and 1440 deaths reported. Some 117 countries/territories/ areas were reporting cases.

http://bit.ly/2QbBSLD

## COVID-19 solidarity fund.

The WHO Director-General announced the creation of the COVID-19 solidarity response fund. Launched 13 March, the fund will become the leading mechanism worldwide for businesses, philanthropies, and individuals to contribute to the COVID-19 response effort.

WHO estimates that US$675 million will needed to fund preparedness and response efforts through April 2020 alone. As the COVID-19 outbreak evolves, funding needs are likely to increase.

Contributions to the fund will play a critical role in country-level support, including: tracking and understanding the spread of the virus, ensuring patients get the care they need, and that frontline workers get the supplies and information they require.

http://bit.ly/33m81Wo

## Shortage of personal protective equipment endangers health workers

Disruption to the global supply of personal protective equipment is putting lives at risk from COVID-19 and other infectious diseases. As of 3 March, WHO had shipped nearly half a million sets of personal protective equipment to 47 countries, but supplies are rapidly diminishing because of rising demand, hoarding and misuse.

Limited access to supplies such as gloves, medical masks, respirators, goggles, face shields, gowns, and aprons exposes doctors, nurses and other frontline workers to infection from COVID-19 and other diseases.

On 3 March WHO issued an appeal for action to address the situation. “Industry and governments must act quickly to boost supply, ease export restrictions and put measures in place to stop speculation and hoarding. We can’t stop COVID-19 without protecting health workers first,” said WHO Director-General Dr Tedros.

http://bit.ly/2v9c1wF

## United Nations funding for COVID-19 response

The Central Emergency Response Fund (CERF) of the United Nations released US$15 million to help fund global efforts to contain the COVID-19 virus. Released 1 March, the funds were sent to WHO and the United Nations Children’s Fund which will use the money to support essential activities including monitoring the spread of the virus, investigating cases, and running national laboratories.

The funds were released on the day WHO upgraded the global risk of the coronavirus outbreak to "very high" – its highest risk assessment level, an assessment based in part on the sharp increase in cases in Italy, the Islamic Republic of Iran and the Republic of Korea.

http://bit.ly/339QQHA

## COVID-19 schools guidance

WHO, the International Federation of the Red Cross (IFRC), and the United Nations Children’s Fund (UNICEF) issued guidance to help protect children and schools from transmission of the COVID-19 virus.

Issued 11 March, the guidance provides practical checklists to help keep schools safe and advises national and local authorities on how to adapt and implement emergency plans for educational facilities.

The guidance includes recommendations to mitigate the possible negative impacts on children’s learning and wellbeing where school closures are implemented. Where schools remain open, the guidance calls for: providing children with information about how to protect themselves; promoting best handwashing and hygiene practices and providing hygiene supplies; cleaning and disinfecting school buildings, especially water and sanitation facilities; and increasing airflow and ventilation.

http://bit.ly/3aNfQGW

## Ebola outbreak winds down

On 3 March the only person confirmed to have Ebola virus disease in the previous 21 days was discharged from an Ebola Treatment Centre in the Democratic Republic of the Congo after recovering and testing negative twice for the virus. This is an important milestone in the outbreak. However, there is still a high risk of re-emergence of the disease and it is vital that response operations be maintained.

Dr Matshidiso Moeti, the World Health Organization Regional Director for Africa, said she was encouraged by the news but stressed the importance of staying vigilant.

Surveillance, pathogen detection and clinical management of patients is ongoing. To provide care for survivors it is essential to sustain the response for at least 18 months after the outbreak is declared over.

The current outbreak, which was declared on 1 August 2018, is the Democratic Republic of the Congo’s tenth and the second-worst globally after the 2014–2016 epidemic in West Africa. As of 10 March, the outbreak had infected 3 444 people (confirmed and probable cases), of whom 2 264 died.

http://bit.ly/2VZDNGW

**Cover Photo**

Artificial intelligence is used to analyse the histological features of some tumours.

**Figure:**
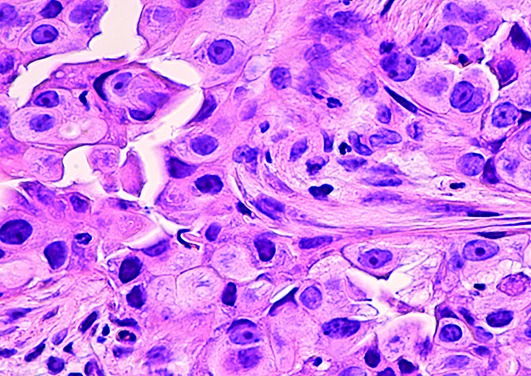
David A. Litman/Shutterstock

## New tuberculosis guidelines

WHO published new recommendations on tuberculosis (TB) preventive treatment. The 18 recommendations cover critical steps in programmatic management that follow the cascade of preventive care. A new regimen of daily rifapentine and isoniazid for one month is conditionally recommended for the first time.

The guidelines are the first to be released under the new consolidated framework that will group all TB recommendations and will be complemented by handbooks with practical advice on how to put in place the recommendations at the scale needed to achieve national and global impact.

http://bit.ly/39KIKHJ

## Women’s health

WHO signed a memorandum of understanding with the Union for the Mediterranean (UfM) to promote and facilitate the engagement of UfM member states in increasing women’s access to health services and in combatting violence against women.

Signed 5 March, the agreement will encourage UfM member states to follow through on commitments they have made in regard to female health and empowerment, notably at the UfM´s 2017 ministerial conference on strengthening the role of women in society, where ministers reaffirmed their commitment to combat all forms of violence against women and girls and to ensure universal access to sexual and reproductive health-care services.

Collaboration between the UfM and WHO started in 2016 with the project “WORTH – Women’s Right to Health,” which aims to reduce the incidence and mortality of cancers affecting women in Albania, Montenegro and Morocco, through the design and implementation of a comprehensive control strategy for cervical and breast cancer.

http://bit.ly/38IWhyj

## France pledges funds for WHO Academy

France pledged US$ 100 million (€90 million) to WHO to support the creation of the WHO Academy, a learning hub that is to be based in Lyon, France.

The pledge was announced by French Minister for Europe and Foreign Affairs, Jean-Yves Le Drian, at a 24 March meeting with WHO Director-General Tedros Adahanom Ghebreyesus.

The WHO Academy aims to reach millions of people worldwide with online and in-person multilingual learning. It will also house a simulation centre for health emergencies.

http://bit.ly/2vn8CKW

**Looking ahead**

23 – 24 April - International Conference on Advanced Health Informatics Tokyo, Japan

17 – 21 May – 73rd session of the World Health Assembly. WHO headquarters. Geneva, Switzerland

